# The Education of the Probationer

**Published:** 1916-07-15

**Authors:** 


### THE MATRONS' AND SISTERS' DEPARTMENT.

### THE EDUCATION OF THE PROBATIONER.

MANY girls who come to hospital for training have had no teaching which will enable them to make notes on the lectures they will attend, nor have they the least idea how to tackle the books to which they may have access so as to really learn anything from them. To these girls lectures, note-taking, and examinations are veritable nightmares. They will spend hours, all their precious "off-duty" times when they ought to be out of doors, trying to "learn up" their lectures from their own illiterate notes (and anyone who has tried to correct these notes or straighten them out knows what a hopeless muddle they are in, in some cases). They may have no more idea at the end of their hours of toil than at the beginning as to what the lecturer really meant or was trying to teach them. But these same girls may be excellent in the wards, observant, quick, skilful, accurate, and thoroughly kind, only through want of proper training they cannot learn easily from books or lectures, or express on paper what they have learnt from practical experience. Of course, there are many others better educated who will profit well by all forms of teaching, but in all hospitals there must be several nurses who want more help than is commonly given.

To begin with, they want teaching how to take notes, not to write down notes from dictation, but to pick out the gist of each sentence as uttered by the lecturer and in a few words make a record which will enable them to reconstruct all that he has said when reading it through afterwards. They also all want instruction in paragraphing and punctuation. And they also need much practice in answering questions, both orally and on paper. "Written orals," as they were called at school, are very useful when the time is limited both for class and correcting, and the pupils not very advanced. The teacher *asks* a question which can be answered in a single sentence, and the pupils *write* their answers. The teacher then immediately gives the correct answer, and the pupils revise their own. A large class can rapidly be questioned in this way, on the whole ground to be covered, and no dull or uninterested person allowed to escape. Probationers also need teaching how to learn from text-books, how to make notes from them and to rapidly assimilate them. This preparatory training in the preliminary school would enable nurses to profit much more from the lectures they hear later from the doctors.

In the wards the sisters are expected to teach the probationers, but how few know how to do it! If they realised the importance of constant questioning of their pupils they would be much more successful. In the unavoidable hard work and rush of a ward so many probationers have not the energy of themselves to take an intellectual interest in their patients' ailments, but if they know "Sister" will be coming round and asking, "Nurse, what is the matter with this patient?" (all probationers do not know what is the matter with the patients to whom they carry dinners and washing-bowls and under whose beds they daily sweep). "What treatment is he having and why? Why is that bed tipped up? Why is that patient on a special diet?" and so forth, they would begin to ask these questions for themselves, and would not be satisfied till they knew all the answers.

Then as to examinations. In some training-schools a nurse may take her "surgical" without having done a day's work in a surgical ward, or her "medical" without any experience of medical work, and she may even, if she is lucky, have polished off her "final" before she has completed her first year! Yet most people surely agree that it is in the wards a nurse really learns her work, and that it takes three years to teach her it, and still she is examined solely on her lectures, and from those, as has already been pointed out, she does not always learn much.

In hospitals where there is a "change" of wards every three months, a short oral or written examination in each ward before the "change" to see what the probationers have managed to learn there would be a great stimulus to both pupils and sisters. The marks gained might be recorded, and would give a fairer estimate of the nurse's progress and zeal than the result of a paper, written in nervous haste, on lectures only half grasped.

Yearly general examinations and a "final" at the end of the three years would keep the nurses up to the mark all through their training. And this "final" would be of much more value if it were largely practical, and if both the practical tests and the papers were set by some central examining body and taken by all training-schools in common, in the same way that our public schools are now examined by the Joint Board for its higher and lower certificates.

